# Paeonol attenuates atherosclerosis by inhibiting mTOR *via* AMPK and SIRT1: Modulating lipid metabolism, autophagy, and pyroptosis

**DOI:** 10.1515/jtim-2026-0041

**Published:** 2026-04-27

**Authors:** Zhi Li, Nan Yan, Xiucong Pei, Xiaoxu Duan, Haijun Tian, Hui Wang, Miao Yu, Jie Ren, Bing Shao

**Affiliations:** Department of Cardiothoracic Surgery, General Hospital of Northern Theater Command, Shenyang, Liaoning Province, China; School of Shuren International, Shenyang Medical College, Shenyang, Liaoning Province, China; School of Public Health, Shenyang Medical College, Shenyang, Liaoning Province, China; School of International Education, Shenyang Medical College, Shenyang, Liaoning Province, China; Department of Cardiology, Second Affiliated Hospital of Shenyang Medical College, Shenyang, Liaoning Province, China

## To the editor

Atherosclerosis (AS) remains a leading cause of cardiovascular mortality globally, driven by lipid metabolism disorders, impaired autophagy, and inflammatory pyroptosis, with unmet clinical needs for therapies targeting its multi-dimensional pathology beyond symptom relief.^[1,2]^ Natural products like paeonol (Pae), a bioactive component from Paeonia suffruticosa, have emerged as promising candidates due to their multitarget effects and favorable safety profiles.^[3,4]^ Here, we report novel mechanistic insights into Pae’s anti-atherosclerotic effects through adenosine-5’-monophosphate-activated protein kinase (AMPK)- and sirtuin 1 (SIRT1)-mediated mammalian target of Rapamycin (mTOR) inhibition, it coordinates lipid metabolism, autophagy, and pyroptosis, supporting its translational and clinical potential for AS management.

Male *ApoE*^–/–^ and C57BL/6J mice were divided into Control (C57BL/6J + normal chow + saline), Control + Pae (C57BL/6J + normal chow + Pae 120 mg/ kg intraperitoneal), AS (*ApoE^–/–^* + high-fat diet [HFD] + saline), and AS + Pae (*ApoE^–/–^* + HFD + Pae) groups, with 11 weeks of intervention after 7 weeks of acclimatization. Key endpoints included atherosclerotic plaque burden (Oil Red O staining), serum lipids, and protein/ gene expression of pathway and functional markers (Western blot, RT-qPCR). All experimental procedures were approved by the Institutional Animal Care and Use Committee (SYYXY2023052001) and complied with ARRIVE guidelines.

**Figure 1:**
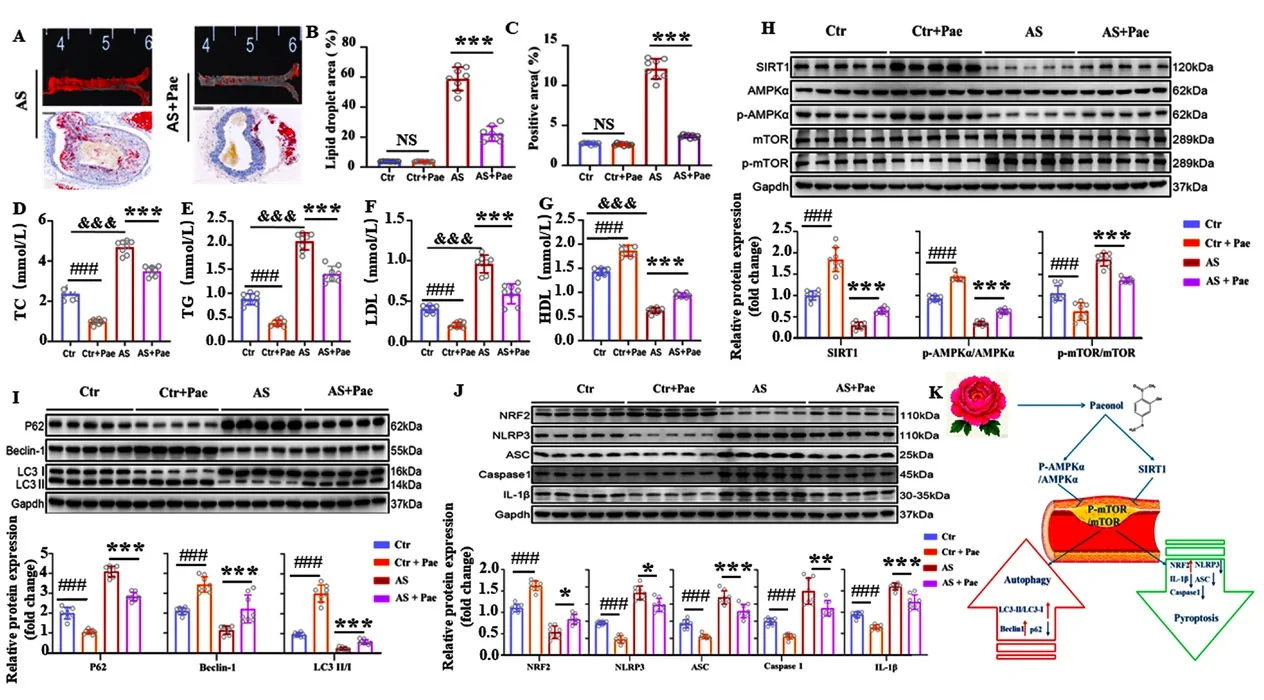
Paeonol alleviates atherosclerosis AS by modulating lipid metabolism, autophagy, and pyroptosis. (A) Representative Oil Red O staining of enface thoracic aorta (full-length aortic flat mount) and cross-sections of aortic root (insets); scale bars: 500 μm (enface), 100 μm (cross-section). (B) Quantification of atherosclerotic lesion area (%) for each group (*n* = 8). (C) Quantification of positive-stained area (%) for each group (*n* = 8). (D-G) Serum lipid profiles: TC, TG, LDL-C, HDL-C (*n* = 8 per group). (H-J) Western blot quantification of target proteins (*n* = 5 per group). (H) Western blot analysis of AMPK, SIRT1 and mTOR protein expression levels in each group (SIRT1, p-AMPKα/total AMPKα, p-mTOR/total mTOR); (I) autophagy markers (LC3-II/LC3-I ratio, Beclin1, p62); (J) pyroptosis-related proteins (NRF2, NLRP3, caspase-1, IL-1β). (K) Mechanistic map of regulating AS by Pae. Data are mean ± SD, analyzed by one-way ANOVA with Tukey’s post-hoc test. ^*^P < 0.05, ^**^P < 0.01, ^***^P < 0.001 (AS group *vs*. AS + Pae group); ^###^*P* < 0.001 (Control group *vs*. Control + Pae group); ^&&&^*P* < 0.001 (AS group *vs*. Control group). Control: C57BL/6J + normal chow + saline; Control + Pae: C57BL/6J + normal chow + Pae; AS: ApoE^–/–^ + high-fat diet + saline; AS + Pae: ApoE^–/–^ + high-fat diet + Pae. Pae: paeonol; AMPK: adenosine-5’-monophosphate-activated protein kinase; SIRT1: sirtuin 1; mTOR: mediated mammalian target of Rapamycin; TC: total cholesterol; TG: triglycerides; LDL-C: low-density lipoprotein cholesterol; HDL-C: high-density lipoprotein cholesterol; p-AMPKα: phosphorylated AMPKα; LC3, microtubule-associated protein light chain 3; NRF2: nuclear factor erythroid-2-related factor 2; NLRP3: nod-like receptor protein 3; IL-1β: interleukin-1β; SD: standard deviation; AS: atherosclerosis.

Pae significantly reduced aortic atherosclerotic plaque area in *ApoE^–/–^* mice (3.64% ± 0.18% *vs*. 12.07% ± 1.26% in AS group, *P* < 0.001; Figure 1A-C) and normalized lipid homeostasis (lowered total cholesterol [TC], triglycerides [TG], low-density lipoprotein cholesterol [LDL-C], elevated high-density lipoprotein cholesterol [HDL-C]; *P* < 0.001 for all; Figure 1D-G). Mechanistically, Pae activated the AMPK/SIRT1 pathway (increased SIRT1 expression and phosphorylated AMPKα [p-AMPKα]/AMPKα ratio) and inhibited mTOR signaling (reduced p-mTOR/mTOR ratio; *P* < 0.001; Figure 1H), which was associated with enhanced autophagy (elevated microtubule-associated protein light chain 3-II [LC3-II]/LC3-I ratio and Beclin1, reduced p62; *P* < 0.001; Figure 1I) and suppressed pyroptosis (downregulated nod-like receptor protein 3 [NLRP3], caspase-1, interleukin-1β [IL-1β] *via* nuclear factor erythroid-2-related factor 2 [NRF2] activation; *P* < 0.05 to *P* < 0.001; Figure 1J). No significant effects on normal lipid homeostasis or vascular physiology were observed in Control + Pae mice, confirming Pae’s safety.

The key novelty lies in Pae’s coordinated regulation of mTOR *via* AMPK and SIRT1, linking lipid metabolism, autophagy, and pyroptosis—three key drivers of AS.^[5,6]^ Unlike statins, which are single-target cholesterol-lowering agents with limited effects on inflammation and autophagy, Pae’s multitarget action addresses the root pathology of AS: its activation of AMPK/SIRT1 promotes fatty acid oxidation and autophagic clearance of lipid droplets, while mTOR inhibition reduces lipogenesis and NLRP3 inflammasome activation *via* NRF2.^[7]^ This network regulation explains Pae’s superior efficacy in plaque reduction and lipid normalization compared to single-pathway interventions.^[8]^

Clinically, Pae’s high vascular bioavailability and low toxicity make it a promising adjunct to statins, especially for statin-intolerant patients or those with residual inflammatory risk. Translational priorities include phase I/II trials to establish human equivalent doses (*via* body surface area scaling^[9]^) and validating p-AMPKα/AMPKα or NRF2 as surrogate biomarkers.^[10]^ Additionally, Pae’s inhibition of NLRP3 inflammasome suggests potential utility in AS comorbidities like metabolic syndrome.

Limitations include the reliance on *ApoE^–/–^* mice (translational gaps to humans) and lack of direct molecular docking data. Future studies should validate these findings in human vascular cells and clinical cohorts.

In conclusion, Pae alleviated AS by coordinating lipid metabolism, autophagy, and pyroptosis *via* inhibiting mTOR through AMPK and SIRT1 (Figure 1K). This work provides a mechanistic framework for developing Pae as a nutraceutical or pharmaceutical agent for AS management, thereby addressing unmet clinical needs in cardiovascular disease.
